# Second-line treatment selection for advanced hepatocellular carcinoma: a network meta-analysis with reconstructed survival curves and integrated benefit–risk assessment

**DOI:** 10.3389/fimmu.2026.1908072

**Published:** 2026-10-08

**Authors:** Xinming Li, Guilin Nie, Yaoqun Wang, Jianrong Xu, Jiaqi Yang, Enyang He, Geng Liu, Bei Li, Jiong Lu

**Affiliations:** 1 Division of Biliary Surgery, Department of General Surgery, West China Hospital of Sichuan University, Chengdu, Sichuan, China; 2 Research Center for Biliary Diseases, West China Hospital, Sichuan University, Chengdu, Sichuan, China

**Keywords:** benefit-risk assessment, hepatocellular carcinoma, network meta-analysis, reconstructed survival data, second-line therapy

## Abstract

**Background:**

Optimal second-line systemic therapy for advanced hepatocellular carcinoma (HCC) remains uncertain. This study compared the efficacy and safety of available second-line treatments after tyrosine kinase inhibitor (TKI) failure and explored treatment strategies after immune checkpoint inhibitor (ICI) exposure.

**Methods:**

Twelve phase III randomized controlled trials (RCTs) were included; 11 contributed to the primary post-TKI network, whereas REACH-2 contributed only to the exploratory alpha-fetoprotein (AFP) ≥400 ng/mL subgroup analysis. Reconstructed individual patient data from published Kaplan–Meier curves were used to generate descriptive pooled survival curves, whereas formal overall-survival (OS) comparisons were based on trial-reported hazard ratios in a frequentist random-effects network meta-analysis (NMA). Safety was evaluated using grade ≥3 adverse events (AEs), and benefit–risk profiles were explored using multidimensional distance to the corner (MDC). Seven observational studies were synthesized separately in an exploratory analysis of post-ICI strategies.

**Results:**

In the random-effects NMA, regorafenib, cabozantinib and pembrolizumab significantly improved OS compared with placebo or best supportive care. No significant OS differences were observed between regorafenib and cabozantinib or pembrolizumab. Among patients with AFP ≥400 ng/mL, ramucirumab was associated with improved OS versus placebo or best supportive care in the exploratory subgroup analysis. Pembrolizumab had the lowest exploratory MDC (0.439), followed by regorafenib (0.693) and cabozantinib (0.908). The MDC ordering remained unchanged across alternative weighting and leave-one-out sensitivity analyses. The direction and statistical significance of the OS estimates were unchanged in the common-effects sensitivity analysis. In exploratory post-ICI analyses, regorafenib combined with ICIs was associated with improved OS compared with regorafenib monotherapy.

**Conclusions:**

After TKI failure, several second-line agents improved OS, with differing safety profiles. Pembrolizumab showed the most favorable integrated efficacy–safety profile among treatments with demonstrated OS benefit and may be particularly relevant when tolerability is a priority, while ramucirumab remains a biomarker-directed option for patients with AFP ≥400 ng/mL. In the exploratory post-ICI analysis, combination therapy with regorafenib and ICI was associated with a potential survival benefit compared with regorafenib alone.

**Systematic Review Registration:**

https://www.crd.york.ac.uk/PROSPERO/view/CRD420261329202, identifier CRD420261329202.

## 1 Introduction

The global incidence of hepatocellular carcinoma (HCC) remains high, and it is one of the leading causes of cancer-related death worldwide (1). Although the therapeutic landscape has expanded over the past decade, most patients eventually experience disease progression after first-line systemic therapy. Therefore, identifying optimal sequential strategies for second-line and subsequent treatments has become a major priority in the clinical management of advanced HCC.

In the pre-immune checkpoint inhibitors (ICIs) era, tyrosine kinase inhibitors (TKIs) such as sorafenib were the cornerstone of first-line therapy for advanced HCC (2). Subsequent randomized controlled trials (RCTs) established a variety of second-line treatment options. However, these trials were conducted in different eras and with different comparator drugs, so a direct head-to-head comparison of these drugs is lacking (3). The relative efficacy and safety of these agents remain uncertain, leaving clinicians with limited guidance when selecting second-line treatments. In recent years, ICI-based combination therapies, such as atezolizumab plus bevacizumab, have become established as first-line standard of care (4). Most evidence regarding second-line therapies was generated in patients who progressed after TKI monotherapy (5). However, data on second-line treatment after ICIs are still lacking and are mainly limited to retrospective or real-world studies (6).

To address these challenges, we conducted a comprehensive evidence synthesis of systemic therapies for the second-line treatment of advanced HCC. Our primary analysis consisted of a random-effects network meta-analysis (NMA) of randomized controlled trials conducted after TKI failure to compare the efficacy and safety of available treatments. We generated pooled descriptive Kaplan–Meier curves using individual patient data (IPD) reconstructed from published curves to visualize survival patterns of different treatments. We also performed an exploratory synthesis of observational evidence to examine treatment strategies in the evolving post-ICI setting. By combining an updated HR-based NMA, descriptive reconstructed survival curves, an exploratory efficacy–safety assessment, and a separate synthesis of post-ICI observational evidence, this study aimed to provide a multidimensional framework to support clinical decision-making in an increasingly complex treatment environment.

## 2 Methods

### 2.1 Reporting standard and protocol registration

This systematic review and network meta-analysis were conducted in accordance with the PRISMA 2020 statement and the PRISMA extension for network meta-analyses (7, 8). The review protocol was prospectively registered in PROSPERO (CRD420261329202; version 1.0 published on 1 March 2026). As the analysis utilized previously published, de-identified aggregate data, ethical approval and informed consent were not required. The registered protocol proposed a Bayesian NMA using reconstructed IPD, credible intervals, and SUCRA. In the final analysis, formal comparative inference was based on trial-reported HRs in a frequentist random-effects NMA with P-scores, while reconstructed IPD was retained for descriptive visualization.

### 2.2 Sources of data and search strategy

We performed a comprehensive literature search across PubMed, Embase, Web of Science, and the Cochrane Central Register of Controlled Trials (CENTRAL) for studies published from January 2010 to December 2025. All databases and other sources were last searched in December 2025. The search strategy employed a combination of Medical Subject Headings (MeSH) and free-text keywords, including “hepatocellular carcinoma,” “advanced/unresectable,” “second-line therapy,” and specific agent names such as regorafenib, cabozantinib, ramucirumab, apatinib, and various ICIs. To capture the most recent evidence, we manually screened abstracts from major international oncology congresses (e.g., ASCO, ESMO) and the reference lists of retrieved primary studies and relevant systematic reviews. The full electronic search strategy is shown in **Supplementary Table S1**.

### 2.3 Study selection and eligibility criteria

Study selection was independently conducted by two reviewers using a two-stage screening process, including title and abstract screening followed by full-text review. Studies were assessed according to predefined eligibility criteria and categorized based on study design and prior systemic therapy exposure. RCTs evaluating systemic therapies primarily in the second-line setting after prior systemic treatment, predominantly TKI therapy, were included in the systematic review. Twelve phase III RCTs comprising 5,505 patients in their full randomized populations were included; 11 contributed to the primary post-TKI network, whereas REACH-2 contributed only to the exploratory AFP ≥400 ng/mL subgroup analysis (9; 4, 10; 11–19). Seven observational studies assessing treatment strategies after ICI exposure were included in a separate exploratory analysis (20–26).

Eligible RCTs enrolled adult patients with advanced or unresectable hepatocellular carcinoma and reported OS HRs with corresponding 95% CIs or sufficient information for their extraction. Availability of a published Kaplan–Meier curve was required only for inclusion in the descriptive reconstructed-curve analysis and was not required for inclusion in the formal NMA. Observational studies were required to report comparative effectiveness between at least two systemic treatment strategies after prior ICI exposure. We excluded studies that evaluated first-line therapy, lacked extractable survival data, or did not include an appropriate comparator for quantitative synthesis. The AHELP trial was excluded because the treatment effect for the target post-TKI population could not be extracted separately (27). Phase II RCTs were also excluded. Cohen’s κ was 0.74 for title and abstract screening and 0.88 for full-text screening; discrepancies were resolved through discussion or, when necessary, adjudication by a third reviewer. The study selection flowchart is shown in **Supplementary Figure S1**.

### 2.4 Data extraction and quality assessment

Data were independently extracted by two reviewers using a standardized electronic form, encompassing study characteristics, patient demographics, analysis populations, potential effect modifiers relevant to transitivity, outcome definitions, effect estimates, and safety-event counts. Any discrepancies were resolved through discussion or, when necessary, consultation with a third reviewer. Baseline characteristics and potential effect modifiers relevant to transitivity are presented in **Supplementary Table S2**.

Methodological quality and risk of bias for RCTs were independently assessed by two reviewers using the Cochrane Risk of Bias 2 (RoB 2) tool (28), covering domains such as the randomization process, deviations from intended interventions, missing outcome data, outcome measurement, and selection of the reported result. Any disagreements were resolved by discussion until consensus was reached, with adjudication by a third reviewer when required. Risk of bias assessments are shown in **Supplementary Table S3**.

### 2.5 Outcomes and clinical endpoints

The primary efficacy endpoint was overall survival (OS), defined as the time from randomization to death from any cause. The secondary endpoint was safety, defined as the proportion of patients experiencing at least one all-cause grade ≥3 adverse event (AE) or treatment-emergent adverse event (TEAE) during the trial safety period. Treatment-related AEs, serious AEs, and treatment-emergent serious AEs were not used as substitutes for this endpoint. Study-specific endpoint definitions, analysis populations, event counts, and data derivation are provided in **Supplementary Table S4B**. Progression-free survival (PFS) was not included because assessment schedules differed across trials and its surrogacy for OS is inconsistent in the second-line setting. Objective response rate, disease control rate, and duration of response were also outside the focused OS and safety scope of this study.

### 2.6 Statistical analysis

To complement the aggregate-data network meta-analysis and to visualize survival patterns over time, we reconstructed individual patient data (IPD) from digitized Kaplan–Meier OS curves using the validated Guyot algorithm (29). The reconstructed IPD were used to generate descriptive pooled Kaplan–Meier curves by treatment. These pooled curves do not preserve randomization across trials and were not used for formal comparative inference or treatment ranking.

For the primary comparative analysis, we conducted a network meta-analysis of randomized controlled trials using hazard ratios (HRs) for OS reported in the original trials, along with their corresponding 95% confidence intervals (CIs). These estimates were synthesized within a frequentist random-effects framework to account for expected clinical and methodological heterogeneity across studies. The exact trial, analysis population, and HR source for each OS input are provided in **Supplementary Table S4A**, and the populations contributing to each analysis are summarized in **Supplementary Table S5**. Cabozantinib was assessed using the CELESTIAL subgroup in which sorafenib was the only prior systemic therapy. Ramucirumab in the primary post-TKI network was assessed using the REACH full ITT population (HR 0.87, 95% CI 0.72–1.05); REACH-2 was not included in the primary network. The resulting network estimates were used to construct the league table summarizing pairwise comparative effects across treatments. Subgroup analyses were exploratory and used only trial-reported subgroup HRs verified against the original reports. In the AFP ≥400 ng/mL analysis, ramucirumab evidence comprised the prespecified REACH AFP-high subgroup and the full REACH-2 ITT population. REACH-2 was excluded from the AFP<400 ng/mL and all non-AFP subgroup analyses. Random-effects models were fitted where between-study heterogeneity was estimable. For AFP<400 ng/mL, each direct comparison was represented by one study and the residual degrees of freedom were zero; heterogeneity was therefore not estimable, τ^2^ was fixed at 0, and common-effects estimates were reported. For safety, eligible study-level event counts were synthesized as odds ratios (ORs) with 95% CIs in a frequentist random-effects NMA using placebo/BSC as the reference; analyses used the eligible trial safety populations, and studies without suitable aggregate counts were excluded. Sources of heterogeneity in the safety network were examined using study-level residual Q contributions and leave-one-study-out sensitivity analyses. Formal meta-regression was not performed because the seven-study network had only one residual degree of freedom, precluding reliable estimation of study-level moderators. No adjustment for multiple comparisons was performed, and all subgroup findings were interpreted cautiously.

Network geometry was visualized using network plots. The post-TKI network was star-shaped and lacked closed loops, meaning local inconsistency and node-splitting could not be evaluated. Transitivity was assessed by comparing key study and patient characteristics across trials (30), including region, treatment line, biomarker selection, ECOG status, viral etiology, tumor burden, and control-arm OS (**Supplementary Table S2**). Funnel plots and Egger’s tests were not performed because most comparisons included too few studies. The post-ICI observational evidence was synthesized separately in a frequentist random-effects network model using study-reported HRs where available. When studies reported pooled treatment categories but provided separate drug-specific Kaplan–Meier curves, drug-specific HRs were estimated from reconstructed survival data and included only in this exploratory analysis. Its single closed loop was evaluated by exploratory node-splitting, but the small number of studies limited statistical power.

Treatment rankings were summarized using frequentist P-scores derived from the complete OS and safety networks (31). Higher P-scores indicate more favorable relative rankings for OS and lower relative odds of grade ≥3 AEs for safety; P-scores are not probabilities of being the best treatment and were interpreted descriptively. An exploratory MDC analysis was performed to integrate the complete-network OS and safety P-scores. MDC candidacy was restricted to treatments demonstrating a statistically significant OS benefit versus placebo/BSC: cabozantinib, pembrolizumab and regorafenib. Treatments without demonstrated OS benefit remained in the complete evidence networks but were excluded from the MDC candidate set. MDC was calculated as


$$\text{MDC}=\sqrt{{\left(1-{\text{P-score}}_{OS}\right)}^{2}+{\left(1-P{\text{-score}}_{AE}\right)}^{2}}$$

Higher P-scores indicate more favorable relative rankings for the corresponding outcome, and a lower MDC indicates a more favorable integrated efficacy–safety position. Because the integrated assessment focused on two prespecified outcomes, OS and grade ≥3 AEs, we selected MDC rather than a broader multicriteria decision analysis framework such as TOPSIS (32, 33). MDC directly summarizes the distance of the two NMA-derived P-scores from an ideal efficacy–safety corner, providing a simple and transparent measure with relatively few additional modelling assumptions. This approach also retains a direct link between the integrated measure and the efficacy and safety rankings generated by the NMA. To examine the influence of criterion weighting, MDC was recalculated using alternative OS-to-safety weights of 2:1 and 1:2, with equal weighting retained as the primary analysis. MDC was also recalculated using safety P-scores from leave-one-study-out analyses excluding KEYNOTE-240 and KEYNOTE-394 separately (**Supplementary Table S7**).

All statistical analyses were performed using R software (version 4.5.2, R Foundation for Statistical Computing, Vienna, Austria). To assess the robustness of the primary efficacy findings to model choice, a common-effects model was fitted as a sensitivity analysis for the post-TKI OS network, while the random-effects model remained the primary analysis (**Supplementary Table S6**). Trial-reported and population-aligned reconstructed HRs were also compared where corresponding Kaplan–Meier curves were available (**Supplementary Figure S7**). All statistical tests were two-sided, and a P value<0.05 was considered statistically significant.

## 3 Results

### 3.1 Characteristics of included studies

The systematic review included 12 phase III RCTs involving 5,505 patients with advanced or unresectable HCC who had received prior systemic therapy, predominantly sorafenib. Of these, 11 contributed to the primary post-TKI network, whereas REACH-2 contributed only to the exploratory AFP ≥400 ng/mL subgroup analysis. These trials evaluated a range of targeted agents and immunotherapies, including regorafenib, cabozantinib, ramucirumab, pembrolizumab, and other investigational agents, most commonly in comparison with placebo or placebo plus best supportive care. In the exploratory analysis, we included 7 observational studies that investigated subsequent systemic treatment strategies after prior ICI-based therapy, most commonly atezolizumab plus bevacizumab. Given differences in study design and evidence level, randomized trials constituted the primary basis for comparative analyses, whereas observational studies were included to provide contextual and exploratory evidence. The literature screening process is shown in **Supplementary Figure S1**.

### 3.2 Second-line therapies after TKI: NMA findings

#### 3.2.1 Overall survival

The primary post-TKI OS network included 11 phase III RCTs involving 5,000 patients with advanced HCC who had received prior systemic therapy, predominantly TKIs. Reconstructed IPD were used to generate descriptive pooled Kaplan–Meier curves (**Figure 1**). The ramucirumab curve was based on the REACH full ITT population, consistent with the population used in the primary post-TKI network. The network followed a star-shaped configuration centered on placebo or best supportive care (BSC), and all active-treatment comparisons were therefore indirect (**Supplementary Figure S2A**).

**Figure 1:**
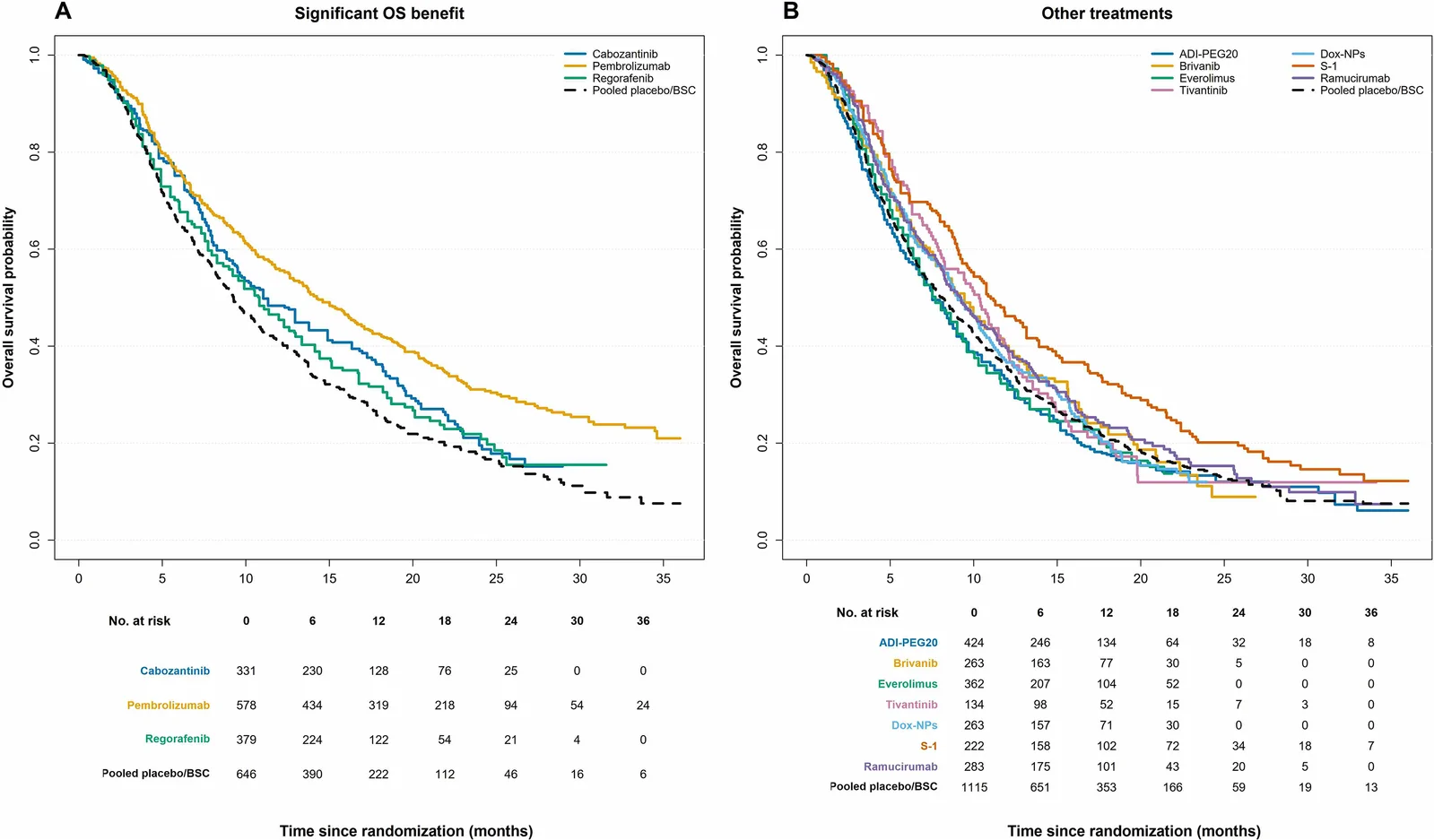
Descriptive pooled reconstructed overall-survival curves. **(A)** shows the three treatments associated with a statistically significant OS benefit and their corresponding pooled placebo/BSC curves. **(B)** shows the other treatment nodes and their corresponding pooled placebo/BSC curves. Ramucirumab was represented by the REACH full ITT population; REACH-2 was not included. Both panels use identical axes and display numbers at risk. The curves were reconstructed from published Kaplan–Meier curves and pooled descriptively by treatment. These curves do not preserve cross-trial randomization and were used only for descriptive visualization, not formal treatment comparison or ranking.

In the descriptive pooled curves, pembrolizumab showed sustained separation from the corresponding pooled placebo/BSC curve during extended follow-up (**Figure 1**). These curves were not used for formal cross-trial comparisons or treatment ranking. The NMA-derived league table showed that regorafenib (HR 0.63, 95% CI 0.50–0.79), cabozantinib (HR 0.70, 95% CI 0.55–0.89), and pembrolizumab (HR 0.79, 95% CI 0.67–0.93) significantly improved OS relative to placebo/BSC, whereas ramucirumab did not show a statistically significant benefit (HR 0.87, 95% CI 0.72–1.05) (**Figure 2**; **Supplementary Figure S3A**). Among the treatments with a significant OS benefit, regorafenib had the highest complete-network OS P-score (0.949), followed by cabozantinib (0.850) and pembrolizumab (0.707) (**Table 1**; **Supplementary Figure S4**). No statistically significant OS differences were observed among regorafenib, cabozantinib, and pembrolizumab (**Figure 2**). OS heterogeneity was low (I^2^ = 0%; τ^2^ = 0). Most trials enrolled predominantly Child–Pugh A patients, but several potential effect modifiers varied across the contributing populations. Among reporting arms, the proportion of patients with ECOG 0 ranged from approximately 39% to 85%, MVI from 11% to 35%, and EHS from 52% to 78%; viral etiology also differed substantially, with HBV predominating in KEYNOTE-394 and HCV in S-CUBE. Control-arm median OS ranged from 7.2 to 13.0 months. Differences in geographic region, prior-treatment eligibility, and biomarker selection were also identified and were considered when interpreting the indirect active-treatment comparisons (**Supplementary Table S2**). Differences in viral etiology, tumor burden, and control-arm OS suggest variation in baseline prognosis across trials and, if associated with treatment-effect modification, could influence the indirect active-treatment estimates.

**Figure 2:**
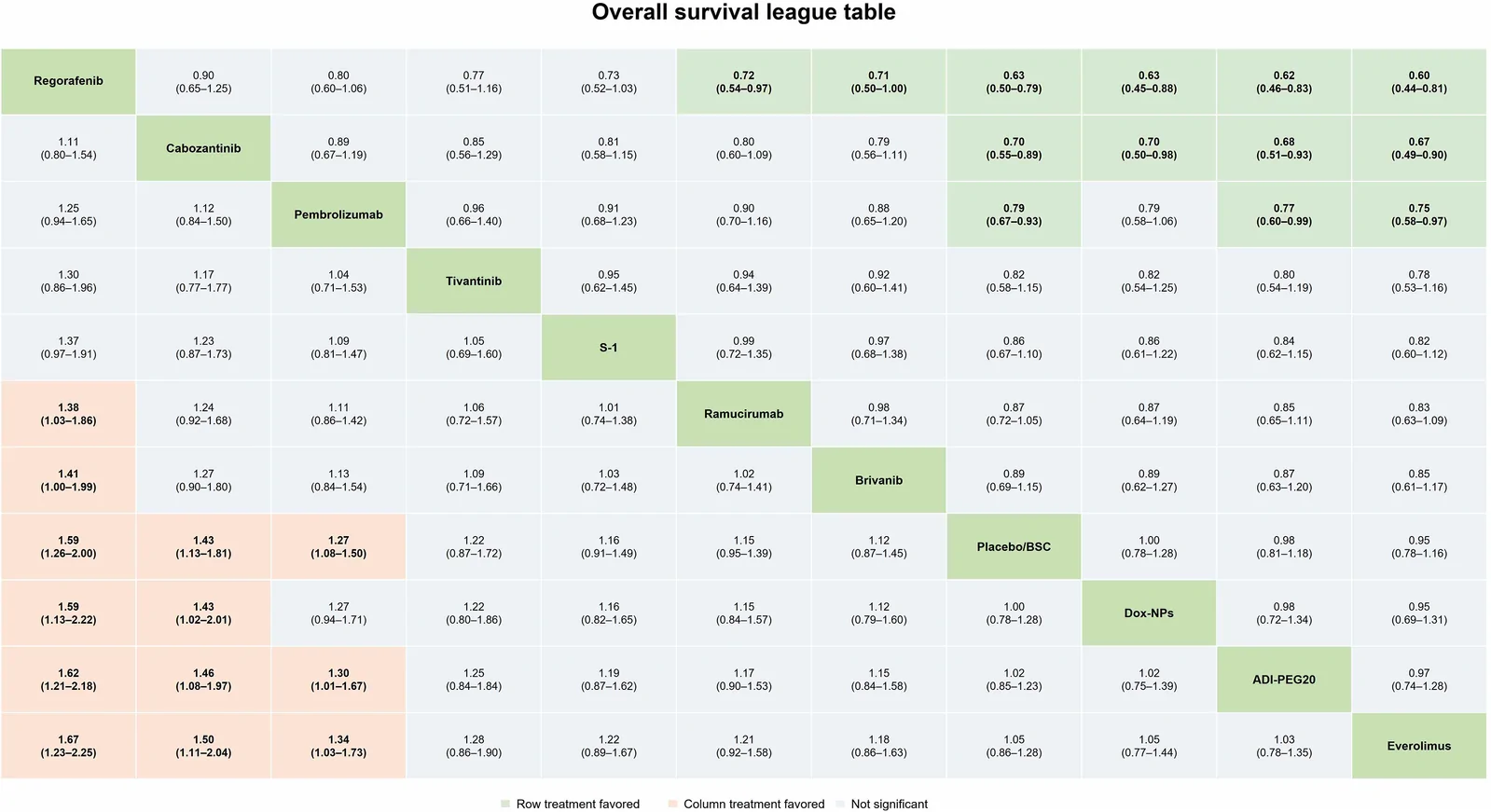
League table showing network estimates for overall survival. Each off-diagonal cell reports the HR with 95% CI for the row treatment versus the column treatment; HR<1 favors the row treatment. The same comparison is displayed in the opposite triangle as its reciprocal. Green cells indicate statistically significant results favoring the row treatment, peach cells indicate statistically significant results favoring the column treatment, and gray cells indicate no statistically significant difference. Significant estimates are shown in bold. Because the network was star-shaped, all active-treatment comparisons were indirect. Statistical significance was determined using unrounded estimates. Ramucirumab was represented by the REACH full ITT population, and REACH-2 was excluded from the primary network.

**Table 1:** Exploratory MDC analysis among treatments demonstrating an overall-survival benefit.

Treatment	Complete-network OS P-score	Complete-network safety P-score	Exploratory MDC
Cabozantinib	0.850	0.104	0.908
Pembrolizumab	0.707	0.673	0.439
Regorafenib	0.949	0.309	0.693

OS and safety P-scores were derived from the complete evidence networks. Higher P-scores indicate more favorable relative rankings, whereas lower MDC values indicate a more favorable efficacy–safety position. The MDC analysis was restricted to treatments with a statistically significant OS benefit versus placebo/BSC and comparable safety data. MDC values were calculated using unrounded full-network P-scores and should be interpreted as exploratory. BSC, best supportive care; MDC, multidimensional distance to the corner; OS, overall survival.

#### 3.2.2 Subgroup analyses

We conducted exploratory subgroup analyses stratified by ECOG performance status, EHS, MVI, viral status, BCLC stage, and AFP levels; the reference treatment varied according to the available network structure (**Supplementary Figures S5, S6A, B**). In the AFP ≥400 ng/mL random-effects analysis, no significant differences were observed between regorafenib and ramucirumab (HR 1.02, 95% CI 0.70–1.47), Dox-NPs (HR 1.32, 95% CI 0.83–2.11), or S-1 (HR 1.50, 95% CI 0.93–2.41), whereas placebo/BSC was associated with poorer OS (HR 1.47, 95% CI 1.08–2.00). For AFP<400 ng/mL, heterogeneity was not estimable because the residual degrees of freedom were zero, and common-effects estimates favored regorafenib over ramucirumab (HR 1.63, 95% CI 1.09–2.42) and Dox-NPs (HR 1.76, 95% CI 1.11–2.79); the comparison with S-1 was not statistically significant (HR 1.18, 95% CI 0.76–1.83), while placebo/BSC was associated with poorer OS (HR 1.49, 95% CI 1.11–2.00). Given the exploratory nature of subgroup analyses, these findings should be interpreted cautiously.

#### 3.2.3 Safety

Eligible all-cause grade ≥3 AE or TEAE data were available from seven RCTs involving 3,413 patients; the corresponding network geometry is shown in **Supplementary Figure S2B**. Compared with placebo/BSC, cabozantinib (OR 4.73, 95% CI 2.16–10.32) and regorafenib (OR 3.10, 95% CI 1.45–6.62) were associated with significantly higher odds of grade ≥3 AEs, whereas the lower estimates for pembrolizumab (OR 1.72, 95% CI 0.99–2.99) and ramucirumab (OR 1.79, 95% CI 0.84–3.78) were not statistically significant (**Figure 3**). Among the three treatments with a significant OS benefit, pembrolizumab had a lower estimated OR than regorafenib and cabozantinib, although its confidence interval included 1. The safety network showed substantial heterogeneity (I^2^ = 72.68%; τ^2^ = 0.1164). Pembrolizumab was the only treatment represented by two trials, and in the residual-Q diagnostic, KEYNOTE-240 and KEYNOTE-394 jointly accounted for all estimable residual heterogeneity, with Q contributions of 50.73% and 49.27%, respectively. Excluding either trial left no residual degrees of freedom, such that τ^2^ and I^2^ were no longer estimable. The pembrolizumab OR was 2.28 (95% CI 1.52–3.42) after excluding KEYNOTE-240 and 1.29 (95% CI 0.86–1.95) after excluding KEYNOTE-394, whereas the point estimates for regorafenib and cabozantinib were unchanged (**Supplementary Table S7**). Detailed primary safety results and data sources are provided in **Figure 3** and **Supplementary Table S4B**.

**Figure 3:**
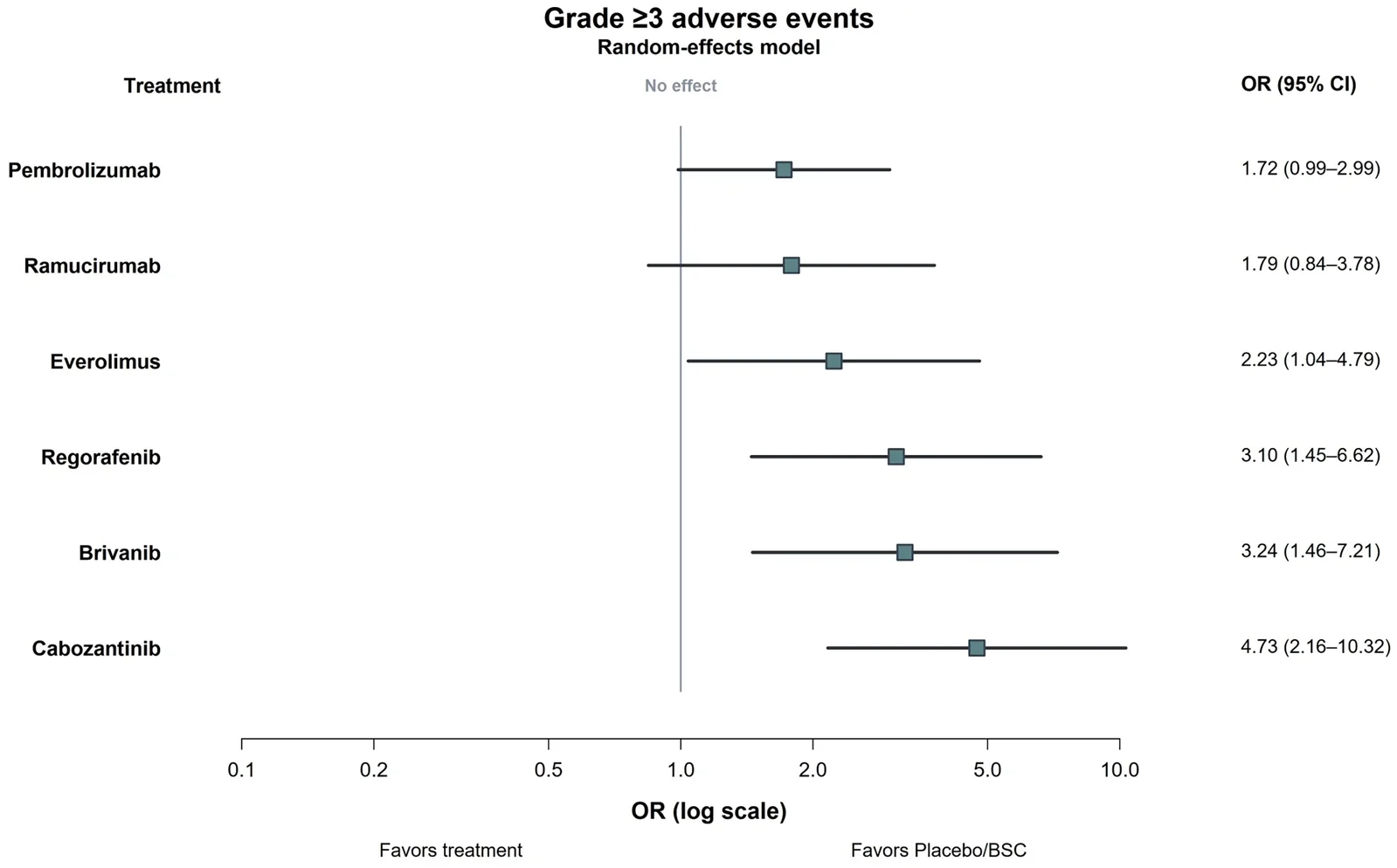
Forest plot of all-cause grade ≥3 adverse events or treatment-emergent adverse events among second-line therapies after prior TKI treatment. The forest plot presents random-effects network ORs with 95% CIs for each listed treatment versus placebo/BSC. OR<1 indicates lower odds of an eligible grade ≥3 event with the listed treatment, whereas OR >1 indicates lower odds with placebo/BSC. Ramucirumab was represented by the REACH full safety population; REACH-2 was not included. Detailed endpoint definitions, event counts, data sources, and derivation methods are provided in **Supplementary Table S4B**.

#### 3.2.4 Exploratory integrated benefit–risk assessment via MDC

Because the three treatments with a significant OS benefit differed in their efficacy and safety rankings, we used MDC to explore their integrated benefit–risk profiles (**Table 1**). Pembrolizumab had the lowest MDC (0.439), followed by regorafenib (0.693) and cabozantinib (0.908). Pembrolizumab therefore had the most favorable exploratory efficacy–safety position among the three treatments. This ordering was unchanged with alternative OS-to-safety weights of 2:1 and 1:2 and after separately excluding KEYNOTE-240 or KEYNOTE-394 from the safety network (**Supplementary Table S7**).

### 3.3 Exploratory synthesis after ICI exposure

We conducted an exploratory network synthesis of seven observational studies evaluating systemic treatment strategies after prior ICI exposure. The corresponding treatment network is shown in **Supplementary Figure S2C**, with regorafenib serving as the main reference node. Regorafenib plus an ICI was associated with improved OS compared with regorafenib alone (HR 0.60, 95% CI 0.37–0.97) (**Supplementary Figure S3B**). Compared with regorafenib, cabozantinib (HR 1.33, 95% CI 0.36–4.97) and lenvatinib (HR 1.44, 95% CI 0.77–2.68) showed no significant OS differences, whereas sorafenib was associated with a higher hazard of death (HR 2.08, 95% CI 1.14–3.79). Given that all included studies were observational, these findings should be considered exploratory and hypothesis-generating.

### 3.4 Risk of bias and robustness of the evidence

Overall risk of bias was low in nine RCTs, while three were judged to have some concerns involving the randomization process, deviations from intended interventions, or selection of the reported result (**Supplementary Table S3**). The post-TKI network contained no closed loops; therefore, local inconsistency could not be assessed. Because estimated between-study heterogeneity was zero (τ^2^ = 0), the common-effects sensitivity analysis yielded estimates identical to those from the primary random-effects OS analysis (**Supplementary Table S6**). The exploratory post-ICI network contained one closed loop, and node-splitting showed no statistically detectable disagreement; however, the small number of studies limited statistical power (**Supplementary Figure S8**).

## 4 Discussion

This NMA compared and explored the efficacy and safety of current second-line systemic therapies for advanced HCC. We used evidence from randomized controlled trials in patients previously treated with TKIs. We also synthesized observational data from patients previously exposed to ICIs in a separate exploratory analysis. Our goal was to inform clinical decision-making in settings where optimal treatment sequencing strategies remain uncertain.

Several recent network meta-analyses (NMAs) have compared the efficacy and safety of second-line therapies for advanced HCC (34–37). Our study extends this evidence in several ways. First, we performed a random-effects NMA using trial-reported hazard ratios (HRs) as the basis for formal treatment comparisons. We also reconstructed individual patient data and generated pooled Kaplan–Meier curves. These curves provide a more intuitive view of how survival patterns change over follow-up. However, they were used only for descriptive visualization and not for formal cross-trial comparisons or treatment ranking. Second, we applied the multidimensional distance to the corner (MDC) among treatments with a demonstrated OS benefit. MDC integrated the OS and safety P-scores derived from grade ≥3 AE data. This provided a single quantitative summary of the trade-off between survival benefit and severe toxicity and allowed a more intuitive comparison of the overall efficacy–safety profiles of the candidate treatments. It may also help place treatment tolerability into the context of individual treatment selection. In addition, previous studies generally did not explicitly separate second-line evidence according to the type of preceding first-line therapy, although most available randomized evidence was generated after TKI treatment. As ICI-based therapy has become increasingly relevant in the first-line setting, we therefore synthesized studies conducted after prior ICI exposure separately to describe the available comparative evidence in this emerging treatment setting.

In the primary post-TKI NMA, regorafenib, cabozantinib, and pembrolizumab significantly improved OS compared with placebo/BSC. In the exploratory AFP ≥400 ng/mL analysis, ramucirumab was associated with improved OS compared with placebo/BSC. The reconstructed curves were used only to visualize pooled survival patterns; the greater separation observed for pembrolizumab should not be interpreted as conflicting with the HR-based findings because these curves reflect cross-trial differences in patient populations, baseline risk, follow-up, and control arms. Among these, regorafenib showed numerically greater improvement in OS. This is consistent with the RESORCE trial and regorafenib’s established role in this treatment setting (10, 38). However, regorafenib was also associated with significantly higher odds of all-cause grade ≥3 AEs (OR 3.10). This toxicity profile may be related to its broad multi-kinase inhibition, targeting pathways including VEGFR, PDGFR, RAF, and other kinases involved in tumor angiogenesis and proliferation (39, 40). Cabozantinib also improved OS but had the highest estimated odds of all-cause grade ≥3 AEs versus placebo/BSC (OR 4.73), highlighting the importance of treatment tolerability. The safety network showed substantial heterogeneity. Formal meta-regression was not performed because only seven studies contributed to the safety network, leaving only one residual degree of freedom after accounting for treatment effects. This was insufficient for reliable estimation of additional study-level moderators. We therefore performed leave-one-study-out analyses. The estimated safety effect and safety rank of pembrolizumab changed when KEYNOTE-240 or KEYNOTE-394 was excluded, indicating some trial-level sensitivity. However, these changes did not alter the subsequent MDC ordering of pembrolizumab, regorafenib, and cabozantinib.

In the exploratory MDC analysis restricted to treatments with a significant OS benefit and eligible safety data, pembrolizumab had the lowest MDC (0.439), followed by regorafenib (0.693) and cabozantinib (0.908). Pembrolizumab’s relatively favorable integrated position may partly reflect its lower estimated odds of grade ≥3 AEs than those of regorafenib and cabozantinib. Pembrolizumab selectively targets programmed cell death protein 1 (PD-1), thereby enhancing T-cell-mediated antitumor activity without the broader kinase inhibition of multi-kinase agents. Under the primary equal-weight MDC specification, pembrolizumab had an MDC 0.254 lower than regorafenib and 0.469 lower than cabozantinib. These findings indicate a consistent separation in the combined efficacy–safety space and support a more favorable integrated profile for pembrolizumab when treatment tolerability is an important consideration. In practice, MDC differences are most useful for comparing the relative separation between candidate treatments: a lower MDC indicates a profile closer to the joint ideal of greater OS benefit and lower severe toxicity, whereas a larger difference indicates greater separation between treatment profiles. Ramucirumab was not included in the MDC analysis because the REACH full ITT population did not meet the OS-benefit criterion, although it remained relevant in the exploratory AFP ≥400 ng/mL analysis.

Most indirect comparisons across the examined subgroups were not statistically significant. HR point estimates for several competing agents were numerically greater than 1.0, although their precision and statistical significance varied across subgroups. In some subgroups characterized by higher tumor burden or poorer prognostic features, such as EHS, MVI, or AFP ≥400 ng/mL, the relative OS differences between regorafenib and other active agents appeared smaller. For example, some comparisons involving ramucirumab or cabozantinib produced HRs closer to 1.0 in these higher-risk subgroups, although this pattern was not consistent across all analyses. These findings raise the possibility that relative treatment differences may be less pronounced in some patients with more advanced disease, but they do not establish a consistent subgroup effect. This observation may partially reflect underlying tumor biology, as advanced disease progression is frequently driven by enhanced VEGF-mediated angiogenesis (41), a pathway targeted by multiple second-line agents. Additionally, elevated AFP levels have been associated with a more immunosuppressive tumor microenvironment (42, 43), potentially reducing response differences between therapies. In the exploratory AFP ≥400 ng/mL analysis, ramucirumab showed an OS estimate similar to regorafenib (HR 1.02, 95% CI 0.70–1.47), and no active comparator differed significantly from regorafenib. This finding is compatible with the biomarker-defined benefit of ramucirumab observed in REACH-2, although that trial compared ramucirumab directly with placebo rather than regorafenib (19). These findings may help contextualize treatment selection but do not establish subgroup-specific superiority, particularly because all active-treatment comparisons were indirect and remained dependent on the transitivity assumption.

These findings also illustrate why treatment selection after prior TKI therapy cannot be determined by efficacy ranking alone. The balance between survival benefit and treatment burden will differ according to hepatic reserve, performance status, comorbidities, previous treatment tolerance, and the feasibility of toxicity monitoring. In this setting, the integrated profile of pembrolizumab may be useful when tolerability carries greater weight in treatment selection. Conversely, the higher OS rankings of regorafenib and cabozantinib may remain clinically relevant for patients who are able to tolerate and manage a greater toxicity burden. AFP status adds another consideration, as randomized evidence supports ramucirumab specifically in patients with AFP ≥400 ng/mL.

Due to the limited evidence guiding subsequent treatment after prior ICI exposure, our study included seven observational studies for analysis. Preliminary results suggested that none of the single-agent regimens demonstrated superior OS compared with regorafenib. However, regorafenib combined with an ICI was associated with improved OS compared with regorafenib alone. In advanced NSCLC, a recent systematic review and single-arm meta-analysis of ICI plus multi-targeted TKI therapy in the second- or later-line setting reported a pooled median PFS of 5.74 months and an ORR of 26.35% (44). Such cross-tumor benefits share a common biological mechanism: multi-targeted TKIs can suppress VEGF-mediated angiogenesis, reverse the immunosuppressive tumor microenvironment (45), and thereby exert synergistic anti-tumor effects with immunotherapy. The safety of this combination strategy requires further evaluation. Because all studies included in this analysis were non-randomized, the estimates may have been affected by differences in patient selection, treatment allocation, and other residual confounding factors. These findings should therefore be considered exploratory, and randomized evidence is needed to clarify treatment sequencing after prior ICI exposure.

Our study has several limitations. First, all active-treatment comparisons were indirect and depended on the transitivity assumption; differences in trial and patient characteristics could not be fully adjusted using aggregate data. Because the randomized network was star-shaped, local inconsistency could not be evaluated. Second, the reconstructed pooled Kaplan–Meier curves did not preserve cross-trial randomization and were used only for descriptive visualization. Third, reporting of all-cause grade ≥3 AEs or TEAEs varied across trials in endpoint definition, ascertainment, and follow-up. Substantial heterogeneity and the possible inclusion of events related to disease progression limit the interpretation of indirect safety comparisons. Because this review focused on OS and grade ≥3 AEs, PFS, objective response rate, disease control rate, and duration of response were not evaluated, limiting the assessment of other aspects of treatment efficacy. Finally, most randomized evidence was generated before the widespread use of ICI-based first-line therapy, while evidence after ICI exposure was limited to observational studies. Thus, inferences from the post-ICI observational synthesis remain descriptive and hypothesis-generating.

## 5 Conclusions

After prior TKI therapy, regorafenib, cabozantinib, and pembrolizumab improved OS compared with placebo/BSC, with differing safety profiles. Among treatments with demonstrated OS benefit, pembrolizumab showed the most favorable integrated efficacy–safety profile and may be particularly suitable when treatment tolerability is a major consideration. Regorafenib and cabozantinib remain reasonable options for patients who can tolerate a greater burden of severe AEs, particularly when greater weight is placed on OS benefit. Therefore, treatment selection should consider efficacy, toxicity, hepatic reserve, performance status, and previous treatment tolerance. For patients with AFP ≥400 ng/mL, ramucirumab provides an additional biomarker-directed option supported by randomized evidence. In the exploratory post-ICI observational analysis, regorafenib plus an ICI was associated with improved OS compared with regorafenib alone.

## Data Availability

The original contributions presented in the study are included in the article/**Supplementary Material**. Further inquiries can be directed to the corresponding author.
