# Supplementary material for: Second-line treatment selection for advanced hepatocellular carcinoma: a network meta-analysis with reconstructed survival curves and integrated benefit–risk assessment
Source: Front Immunol. 2026 Oct 8;17:1908072. doi: 10.3389/fimmu.2026.1908072 (PMC13687082; doi:10.3389/fimmu.2026.1908072)
Supplement: Supplementary file 1 [file DataSheet1.docx]

**Table S1. Literature search strategy.**

| **Database** | **Search strategy** | **Records retrieved** |
| --- | --- | --- |
| PubMed | ("Carcinoma, Hepatocellular"[Mesh]OR hepatocellular carcinoma[tiab]OR HCC[tiab]OR "liver cancer"[tiab])AND(advanced[tiab]OR unresectable[tiab]OR metastatic[tiab])AND(second line[tiab]OR second-line[tiab]OR subsequent[tiab]OR salvage[tiab]OR refractory[tiab]OR "previously treated"[tiab]OR "after sorafenib"[tiab])AND(systemic therap*[tiab]OR targeted therap*[tiab]OR immunotherap*[tiab]OR "tyrosine kinase inhibitor*"[tiab]OR TKI[tiab]OR "immune checkpoint inhibitor*"[tiab]OR ICI[tiab])NOT(review[pt]OR meta-analysis[pt]) | 507 |
| Cochrane | (hepatocellular carcinoma OR HCC OR "liver cancer") AND (advanced OR unresectable OR metastatic) AND ("second line" OR "second-line" OR "2nd line" OR "post-progression" OR regorafenib OR cabozantinib OR ramucirumab OR pembrolizumab) | 424 |
| Embase | (('hepatocellular carcinoma'/exp OR hepatocellular) AND carcinoma:ti,ab OR hcc:ti,ab OR 'liver cancer':ti,ab) AND (advanced:ti,ab OR unresectable:ti,ab OR metastatic:ti,ab) AND ('second line':ti,ab OR subsequent:ti,ab OR salvage:ti,ab OR refractory:ti,ab OR 'previously treated':ti,ab OR 'after sorafenib':ti,ab) AND ('systemic therapy':ti,ab OR 'targeted therapy':ti,ab OR immunotherapy:ti,ab OR 'tyrosine kinase inhibitor':ti,ab OR tki:ti,ab OR 'immune checkpoint inhibitor':ti,ab OR ici:ti,ab) NOT ('review'/it OR 'meta analysis'/it) | 1302 |
| Web of Science | ((hepatocellular carcinoma OR HCC OR "liver cancer") AND (advanced OR unresectable OR metastatic) AND ("second line" OR second-line OR subsequent OR salvage OR refractory OR "previously treated" OR "after sorafenib") AND ("systemic therapy" OR "targeted therapy" OR immunotherapy OR "tyrosine kinase inhibitor" OR TKI OR "immune checkpoint inhibitor" OR ICI)) NOT (review OR "meta-analysis") | 550 |

Note: Search strategies are reproduced as executed in each database. The searches identified 2,783 records in total.

**Figure S1. PRISMA 2020 flow diagram.**

Note: The systematic search identified 2,783 records. After duplicate removal and eligibility screening, 19 studies were included: 12 RCTs and 7 observational studies. Twelve RCTs were included; 11 contributed to the primary post-TKI network, whereas REACH-2 contributed only to the exploratory AFP ≥400 ng/mL subgroup analysis.

**Table S2. Study- and arm-level characteristics and potential effect modifiers relevant to transitivity.**

| Study / trial (first author, year) | Arm | N | Recruitment period | Country/region | Analysis population / prior therapy | Biomarker selection | Child–Pugh A, n (%) | ECOG 0, n (%) | HBV, n (%) | HCV, n (%) | AFP ≥400 ng/mL, n (%) | MVI, n (%) | EHS, n (%) | Control-arm median OS, months | Key transitivity issue |
| --- | --- | --- | --- | --- | --- | --- | --- | --- | --- | --- | --- | --- | --- | --- | --- |
| Randomized post-TKI studies | | | | | | | | | | | | | | | |
| ADI-PEG20 (Abou-Alfa, 2018) | ADI-PEG20 | 424 | NR | Multinational | ITT; ≥1 prior systemic therapy; ≥2L | None | 387(91.3%) | NR | 226(53%) | 112(26%) | NR | NR | NR | 7.4 | Mixed prior therapy; ≥2L |
|  | placebo/BSC | 211 |  |  |  |  | 188(89.1%) | NR | 106(50%) | 55(26%) | NR | NR | NR |  |  |
| BRISK-PS (Llovet, 2013) | Brivanib | 263 | NR | Multinational | ITT; post-sorafenib; 2L | None | 242(92.0%) | 151(57.4%) | 102(39%) | 73(28%) | NR | 81(30.8%) | NR | 8.2 | Refractory/intolerant population |
|  | placebo/BSC | 132 |  |  |  |  | 120(90.9%) | 81(61.4%) | 45(34%) | 35(27%) | NR | 24(18.2%) | NR |  |  |
| CELESTIAL sorafenib-only subgroup (Kelley, 2020) | Cabozantinib | 331 | 2013–2017 | Multinational | Sorafenib-only subgroup | None | 331(100.0%) | 186(56.2%) | 125(38%) | 81(24%) | 129(39%) | 84(25.4%) | 255(77.0%) | 7.2 | Sorafenib-only subgroup selected to align with the post-TKI network |
|  | placebo/BSC | 164 |  |  |  |  | 164(100.0%) | 96(58.5%) | 60(37%) | 43(26%) | 73(45%) | 61(37.2%) | 122(74.4%) |  |  |
| EVOLVE-1 (Zhu, 2014) | Everolimus | 362 | 2010–2012 | Multinational | ITT; post-sorafenib; ≥2L | None | 354(97.8%) | 214(59.1%) | 91(25.1%) | 94(26.0%) | NR | 119(32.9%) | 269(74.3%) | 7.3 | Mixed treatment lines |
|  | placebo/BSC | 184 |  |  |  |  | 182(98.9%) | 104(56.5%) | 52 (28.3%) | 43(23.4%) | NR | 60(32.6%) | 135(73.4%) |  |  |
| JET-HCC(Kudo,2020) | Tivantinib | 134 | 2014–2016 | Japan | MET-high ITT; post-sorafenib; 2L | MET-high | 130(97.0%) | 111(82.8%) | 35(26.1%) | 61(45.5%) | NR | 100(74.6%) | NR | 8.5 | Japan-only; MET-high |
|  | placebo/BSC | 61 |  |  |  |  | 59(96.7%) | 51(83.6%) | 16(26.2%) | 31(50.8%) | NR | 44(72.1%) | NR |  |  |
| KEYNOTE-240 (Finn, 2020) | Pembrolizumab | 278 | 2016–2017 | Multinational | Global ITT; post-sorafenib; 2L | None | 277(99.6%) | 162(58.3%) | 72(25.9%) | 43(15.5%) | NR | 36(12.9%) | 195(70.1%) | 10.6 | No major issue identified |
|  | placebo/BSC | 135 |  |  |  |  | 133(98.5%) | 71(52.6%) | 29(21.5%) | 21(15.6%) | NR | 16(11.9%) | 93(68.9%) |  |  |
| KEYNOTE-394 (Qin, 2023) | Pembrolizumab | 300 | 2017–2019 | Asia | Asian ITT; prior sorafenib/chemotherapy; 2L | None | 300(100.0%) | 124(41.3%) | 236(78.7%) | 5(1.7%) | NR | 33(11.0%) | 232(77.3%) | 13.0 | Asia-only; prior therapy not restricted to TKI |
|  | placebo/BSC | 153 |  |  |  |  | 153(100.0%) | 60(39.2%) | 124(81.0%) | 1(0.7%) | NR | 17(11.1%) | 120(78.4%) |  |  |
| REACH (Zhu, 2015) | Ramucirumab | 283 | 2010–2013 | Multinational | Full ITT; post-sorafenib; 2L | None | 277(97.9%) | 159(56.2%) | 100(35%) | 77(27%) | 119(42%) | 82(29.0%) | 207(73.1%) | 7.6 | Full ITT; biomarker-unselected |
|  | placebo/BSC | 282 |  |  |  |  | 276(97.9%) | 153(54.3%) | 101(36%) | 77(27%) | 131(46%) | 79(28.0%) | 200(70.9%) |  |  |
| REACH-2 (Zhu, 2019) | Ramucirumab | 197 | 2015–2017 | Multinational | AFP ≥400 ITT; post-sorafenib; 2L | AFP ≥400 ng/mL | 197(100.0%) | 113(57.4%) | 71(36%) | 48(24%) | 197(100%) | 70(35.5%) | 141(71.6%) | 7.3 | AFP-selected; subgroup analysis only |
|  | placebo/BSC | 95 |  |  |  |  | 95(100.0%) | 55(57.9%) | 36(38%) | 28(29%) | 95(100%) | 33(34.7%) | 70(73.7%) |  |  |
| RELIVE (Merle, 2019) | doxorubicin-loaded nanoparticles | 263 | 2012–2017 | Multinational | ITT efficacy population; post-sorafenib; ≥2L | None | 223(84.8%) | 150(57.0%) | 24(9%) | 79(30%) | 108(41%) | 92(35.0%) | 152(57.8%) | 9.0 | Mixed treatment lines |
|  | placebo/BSC | 134 |  |  |  |  | 114(85.1%) | 70(52.2%) | 13(10%) | 38(28%) | 63(47%) | 46(34.3%) | 83(61.9%) |  |  |
| RESORCE (Bruix, 2017) | Regorafenib | 379 | 2013–2015 | Multinational | ITT; progression on sorafenib; 2L | None | 373(98.4%) | 247(65.2%) | 143(38%) | 78(21%) | 162(43%) | 110(29.0%) | 265(69.9%) | 7.8 | Required progression on tolerated sorafenib |
|  | placebo/BSC | 194 |  |  |  |  | 188(96.9%) | 130(67.0%) | 73(38%) | 41(21%) | 87(45%) | 54(27.8%) | 147(75.8%) |  |  |
| S-CUBE (Kudo, 2017) | S-1 | 222 | 2009–2012 | Multinational | Full analysis set; post-sorafenib; 2L | None | 180(81.1%) | 188(84.7%) | 44(20%) | 121(55%) | 91(41%) | 36(16.2%) | 121(54.5%) | 11.2 | Asia-only; full analysis set |
|  | placebo/BSC | 111 |  |  |  |  | 90(81.1%) | 90(81.1%) | 26(23%) | 59(53%) | 49(44%) | 23(20.7%) | 58(52.3%) |  |  |

**Table S2. Continued**

| Observational post-ICI studies | | | | | | | | | | | | | | | |
| --- | --- | --- | --- | --- | --- | --- | --- | --- | --- | --- | --- | --- | --- | --- | --- |
| Falette-Puisieux et al., 2023 | Regorafenib | 29 | NR | France | Post-ICI observational cohort; line NR | NR | 27(93.1%) | 1(3.4%) | 9(31.0%) | 3(10.3%) | NR | 8(27.6%) | 25(86.2%) | NR | Observational; residual confounding |
|  | TKIs | 53 |  |  |  |  | 41(77.4%) | 5(9.4%) | 12(22.6%) | 3(5.7%) | NR | 23(43.4%) | 41(77.4%) |  |  |
| Ma et al., 2025 | Regorafenib | 59 | NR | China | Post-ICI observational cohort; line NR | NR | 31(52.5%) | 20(33.9%) | 53(89.8%) | 6(10.2%) | 24(40.7%) | 25(42.4%) | 23(39.0%) | NR | Observational; residual confounding |
|  | Regorafenib+ICI | 103 |  |  |  |  | 54(52.4%) | 34(33.0%) | 91(88.3%) | 12(11.7%) | 35(34.0%) | 54(52.4%) | 49(47.6%) |  |  |
| Cheng et al., 2025 | Regorafenib | 31 | NR | China | Post-ICI observational cohort; line NR | NR | 19(61.3%) | 2(6.5%) | 28(90.3%) | NR | 13(41.9%) | NR | NR | NR | Observational; residual confounding |
|  | Regorafenib+ICI | 67 |  |  |  |  | 51(76.1%) | 5(7.5%) | 59(88.1%) | NR | 24(35.8%) | NR | NR |  |  |
| Persano et al., 2025 | Sorafenib | 51 | NR | Italy, Germany, Portugal, Japan, and South Korea | Post-ICI observational cohort; line NR | NR | 48(94.1%) | NR | 16(31.4%) | 10(19.6%) | 16(31.4%) | NR | 19(37.3%) | NR | Observational; residual confounding |
|  | Lenvatinib | 86 |  |  |  |  | 85(98.8%) | NR | 19(22.1%) | 24(27.9%) | 32(37.2%) | NR | 38(44.2%) |  |  |
| Lombardi et al., 2025 | Lenvatinib | 59 | NR | Multinational (Europe, the USA, and Asia) | Post-ICI observational cohort; line NR | NR | NR | 27(45.8%) | NR | NR | 23(39.0%) | NR | 34(57.6%) | NR | Observational; residual confounding |
|  | Sorafenib | 83 |  |  |  |  | NR | 23(27.7%) | NR | NR | 31(37.3%) | NR | 46(55.4%) |  |  |
| Chon et al., 2024 | Lenvatinib | 36 | NR | S. Korea | Post-ICI observational cohort; line NR | NR | 33(91.7%) | 23(63.9%) | NR | NR | NR | 14(38.9%) | 27(75.0%) | NR | Observational; residual confounding |
|  | Sorafenib | 36 |  |  |  |  | 32(88.9%) | 31(86.1%) | NR | NR | NR | 16(44.4%) | 29(80.6%) |  |  |
| Marell et al., 2025 | Anti-VEGF | 84 | NR | USA | Post-ICI observational cohort; line NR | NR | 38(45.2%) | NR | NR | NR | NR | NR | NR | NR | Observational; residual confounding |
|  | ICI | 23 |  |  |  |  | 16(69.6%) | NR | NR | NR | NR | NR | NR |  |  |

Note: Values are presented as n (%) unless otherwise indicated. Each treatment arm is shown in a separate row, whereas study-level information shared by both arms is displayed once using vertically merged cells. Viral etiology and AFP data were reproduced according to the definitions and analysis populations reported in the original studies; etiologic categories may not be mutually exclusive, and percentages may therefore exceed 100% in some studies. For randomized post-TKI studies, the AFP column refers to AFP ≥400 ng/mL. In the post-ICI observational studies, the available source data were recorded as AFP >400 ng/mL and are reproduced as reported. CELESTIAL data correspond to the subgroup in which sorafenib was the only prior systemic therapy. REACH data correspond to the full intention-to-treat population, whereas REACH-2 included only patients with AFP ≥400 ng/mL and was used only in the AFP ≥400 ng/mL subgroup analysis. Control-arm median OS is reported once for each study. NR indicates not reported. AFP, alpha-fetoprotein; BSC, best supportive care; ECOG, Eastern Cooperative Oncology Group performance status; EHS, extrahepatic spread; HBV, hepatitis B virus; HCV, hepatitis C virus; ICI, immune checkpoint inhibitor; ITT, intention-to-treat; MVI, macrovascular invasion; OS, overall survival; TKI, tyrosine kinase inhibitor; 2L, second-line treatment; ≥2L, second- or later-line treatment.

**Figure S2. Evidence networks.**

Note: A, Overall-survival network for randomized treatments after prior TKI therapy. B, All-cause grade ≥3 AE/TEAE network after prior TKI therapy. C, Exploratory overall-survival network for treatments after prior ICI therapy. Nodes represent treatments, edges represent direct comparisons, and edge width and adjacent numerals indicate the number of contributing studies. The analysis in C is based on observational evidence and should be interpreted as exploratory. AE, adverse event; BSC, best supportive care; ICI, immune checkpoint inhibitor; OS, overall survival; TEAE, treatment-emergent adverse event; TKI, tyrosine kinase inhibitor.

**Figure S3. Overall survival after prior TKI or ICI therapy.**

Note: A, Random-effects network estimates relative to placebo/BSC after prior TKI therapy. B, Exploratory observational network estimates relative to regorafenib after prior ICI therapy. HRs are presented with 95% CIs. HR <1 favors the listed treatment over the respective reference. The analysis in A is based on randomized evidence; the analysis in B is observational and should not be interpreted causally. In panel B, some drug-specific HRs were reconstructed from published Kaplan–Meier curves rather than directly reported. BSC, best supportive care; CI, confidence interval; HR, hazard ratio; ICI, immune checkpoint inhibitor; OS, overall survival; TKI, tyrosine kinase inhibitor.

**Figure S4. Overall-survival and safety P-scores.**

Note: Blue circles indicate overall-survival P-scores, and gray triangles indicate safety P-scores for patients experiencing at least one all-cause grade ≥3 AE/TEAE. Higher overall-survival P-scores indicate more favorable relative survival rankings, whereas higher safety P-scores indicate lower relative odds of grade ≥3 events. Safety P-scores are shown only for treatments with eligible data in the updated safety network. Because the efficacy and safety networks differed in included treatments and analysis populations, the two sets of P-scores are endpoint-specific relative rankings. P-scores are not probabilities of being the best treatment and should not be interpreted as treatment recommendations. AE, adverse event; OS, overall survival; TEAE, treatment-emergent adverse event.

**Figure S5. Subgroup evidence networks.**

Note: A1–F2 show ECOG PS 0/1, HBV/HCV, MVI absent/present, EHS absent/present, BCLC B/C, and AFP ≥400/<400 ng/mL, respectively. Nodes are treatments; edges are direct comparisons; edge width and numerals indicate contributing studies. Only trial-reported subgroup HRs were used, without overall-HR imputation. In F1, ramucirumab evidence comprised the prespecified REACH AFP ≥400 ng/mL subgroup and REACH-2 full ITT; REACH full ITT was not used. REACH-2 was excluded from F2 and all non-AFP networks. AFP, alpha-fetoprotein; BCLC, Barcelona Clinic Liver Cancer; ECOG PS, Eastern Cooperative Oncology Group performance status; EHS, extrahepatic spread; HBV, hepatitis B virus; HCV, hepatitis C virus; HR, hazard ratio; ITT, intention-to-treat; MVI, macrovascular invasion.

**Figure S6A. Subgroup overall-survival estimates: ECOG PS, viral etiology, and MVI.**

**Figure S6B. Subgroup overall-survival estimates: EHS, BCLC stage, and AFP.**

Note: HRs are presented with 95% CIs. D1–D2 and F1–F2 use regorafenib as the reference; E1–E2 use placebo/BSC as the reference. Random-effects estimates are shown for D1–F1. In F2 (AFP <400 ng/mL), each direct comparison was represented by one study, leaving residual df=0; heterogeneity was therefore not estimable, τ² was fixed at 0, and common-effect estimates are shown. HR <1 favors the listed treatment over the stated reference. Only trial-reported subgroup HRs were used. In F1, REACH contributed its prespecified AFP-high subgroup HR and REACH-2 contributed its full-ITT HR; REACH full ITT was not included. REACH-2 was excluded from F2 and all non-AFP analyses. For AFP ≥400 ng/mL, Dox-NPs versus regorafenib was HR 1.32 (95% CI 0.83–2.11), which was not statistically significant. AFP, alpha-fetoprotein; BCLC, Barcelona Clinic Liver Cancer; BSC, best supportive care; CI, confidence interval; ECOG PS, Eastern Cooperative Oncology Group performance status; EHS, extrahepatic spread; HBV, hepatitis B virus; HCV, hepatitis C virus; HR, hazard ratio; ITT, intention-to-treat; MVI, macrovascular invasion.

**Table S3. Risk-of-bias assessment of randomized post-TKI trials included in the systematic review.**

| Study / trial (first author, year) | Bias arising from the randomization process | Bias due to deviations from intended interventions | Bias due to missing outcome data | Bias in measurement of the outcome | Bias in selection of the reported result | Overall RoB |
| --- | --- | --- | --- | --- | --- | --- |
| ADI-PEG20 (Abou-Alfa, 2018) | Some concerns | Low risk | Low risk | Low risk | Low risk | Some concerns |
| BRISK-PS (Llovet, 2013) | Low risk | Low risk | Low risk | Low risk | Low risk | Low risk |
| CELESTIAL sorafenib-only subgroup (Kelley, 2020) | Low risk | Low risk | Low risk | Low risk | Low risk | Low risk |
| EVOLVE-1 (Zhu, 2014) | Low risk | Low risk | Low risk | Low risk | Low risk | Low risk |
| JET-HCC (Kudo, 2020) | Low risk | Low risk | Low risk | Low risk | Some concerns | Some concerns |
| KEYNOTE-240 (Finn, 2020) | Low risk | Low risk | Low risk | Low risk | Low risk | Low risk |
| KEYNOTE-394 (Qin, 2023) | Low risk | Low risk | Low risk | Low risk | Low risk | Low risk |
| REACH (Zhu, 2015) | Low risk | Some concerns | Low risk | Low risk | Low risk | Some concerns |
| REACH-2 (Zhu, 2019) | Low risk | Low risk | Low risk | Low risk | Low risk | Low risk |
| RELIVE (Merle, 2019) | Low risk | Low risk | Low risk | Low risk | Low risk | Low risk |
| RESORCE (Bruix, 2017) | Low risk | Low risk | Low risk | Low risk | Low risk | Low risk |
| S-CUBE (Kudo, 2017) | Low risk | Low risk | Low risk | Low risk | Low risk | Low risk |

Note: Risk of bias was assessed using the Cochrane Risk of Bias 2 tool across five domains. Overall judgments were assigned according to the RoB 2 decision algorithm. REACH-2 is retained as an included RCT and contributed only to the exploratory AFP ≥400 ng/mL subgroup analysis.

**Table S4A. Sources of overall survival hazard ratios used in the randomized network meta-analysis.**

| Study | Treatment | Analysis population | HR (95% CI) | Source |
| --- | --- | --- | --- | --- |
| ADI-PEG20 | ADI-PEG20 | Phase III ITT population | 1.022 (0.847–1.233) | Abou-Alfa et al. 2018 |
| BRISK-PS | Brivanib | Sorafenib-failed/intolerant ITT population | 0.89 (0.69–1.15) | Llovet et al. 2013 |
| CELESTIAL sorafenib-only subgroup* | Cabozantinib | Sorafenib-only subgroup (331/164) | 0.70 (0.55–0.88) | Kelley et al. 2020 |
| EVOLVE-1 | Everolimus | Phase III ITT population after sorafenib | 1.05 (0.86–1.27) | Zhu et al. 2014 |
| JET-HCC | Tivantinib | Japanese MET-high ITT population | 0.82 (0.58–1.15) | Kudo et al. 2020 |
| KEYNOTE-240 | Pembrolizumab | Global phase III ITT population | 0.781 (0.611–0.998) | Finn et al. 2020 |
| KEYNOTE-394 | Pembrolizumab | Asian phase III ITT population | 0.79 (0.63–0.99) | Qin et al. 2023 |
| REACH | Ramucirumab | Full ITT population (283/282) | 0.87 (0.72–1.05) | Zhu et al. 2015 |
| RELIVE | Dox-NPs | Phase III ITT efficacy population | 1.00 (0.78–1.28) | Merle et al. 2019 |
| RESORCE | Regorafenib | ITT population after sorafenib progression | 0.63 (0.50–0.79) | Bruix et al. 2017 |
| S-CUBE | S-1 | Full analysis set (222/111) | 0.86 (0.67–1.10) | Kudo et al. 2017 |

Note: Trial-reported HRs were used in the primary random-effects NMA. * Published HR for the subgroup in which sorafenib was the only prior systemic therapy. Ramucirumab in the primary network used REACH full ITT. For the exploratory AFP ≥400 ng/mL subgroup analysis, REACH contributed its prespecified AFP-high subgroup HR and REACH-2 contributed its full-ITT HR; overall HRs were not used to fill missing subgroup estimates. Reconstructed HRs were used only for descriptive source comparison and KM visualization and did not enter the NMA, P-score calculation, or treatment ranking. CI, confidence interval; HR, hazard ratio; ITT, intention-to-treat; NMA, network meta-analysis.

**Table S4B. Sources of all-cause grade ≥3 adverse-event counts used in the safety network meta-analysis.**

| Study | Population | Source endpoint | Active e/n | Control e/n | Data handling | Source |
| --- | --- | --- | --- | --- | --- | --- |
| KEYNOTE-240 | As-treated population | All-cause grade ≥3 AEs | 147/279 | 62/134 | Directly reported | Main article, Table 3 |
| KEYNOTE-394 | Safety population | All-cause grade ≥3 AEs | 157/299 | 50/153 | Sum of grades 3–5 | Main article, Safety section |
| BRISK-PS | Safety population | All-cause grade 3/4 AEs | 188/261 | 58/131 | Rounded estimate | Appendix, Table A3 |
| CELESTIAL sorafenib-only subgroup | Sorafenib-only safety subgroup | All-cause grade 3/4 AEs | 224/329 | 51/164 | Directly reported | Kelley 2020 subgroup analysis, Table 3 |
| EVOLVE-1 | Safety population | All-cause grade 3/4 AEs | 256/361 | 95/182 | Sum of grades 3 and 4 | Main article, Table 3 |
| RELIVE | Safety population | Aggregate grade ≥3 AEs | / | / | Unavailable | Main article, Table 3 |
| RESORCE | Safety population | Grade 3/4 TEAEs | 248/374 | 75/193 | Sum of grades 3 and 4 | Main article, Table 3 |
| REACH | Full safety population | Grade ≥3 TEAEs | 172/277 | 132/276 | Directly reported | Supplementary Appendix, Table S5 |

Note: The target safety outcome was the number of patients experiencing at least one all-cause grade ≥3 AE or TEAE during the trial safety period, irrespective of causality. Treatment-related AEs, serious AEs, and treatment-emergent serious AEs were not used as substitutes. KEYNOTE-394 counts were derived by summing mutually exclusive maximum-grade categories, and BRISK-PS counts were estimated from reported percentages. RELIVE was excluded because no eligible aggregate count was available. REACH used the full safety population, whereas REACH-2 was not included in the safety network. AE, adverse event; e, patients with an event; n, safety population; SAE, serious adverse event; TEAE, treatment-emergent adverse event.

**Table S5. Study-specific populations contributing to each analysis.**

| Study | Randomized N | OS N | KM N | Grade ≥3 AE N | MDC–OS N | MDC–AE N | Remarks |
| --- | --- | --- | --- | --- | --- | --- | --- |
| ADI-PEG20 | 635 | 635 | 635 | — | — | — | No eligible grade ≥3 AE count. |
| BRISK-PS | 395 | 395 | 395 | 392 | — | — | AE count estimated from rounded percentages. |
| CELESTIAL | 707 | 495 | 495 | 493 | 495 | 493 | Sorafenib-only subgroup used to align with the post-TKI population. |
| EVOLVE-1 | 546 | 546 | 546 | 543 | — | — | Safety N excludes 3 untreated patients. |
| JET-HCC | 195 | 195 | 195 | — | — | — | No eligible grade ≥3 AE count. |
| KEYNOTE-240 | 413 | 413 | 413 | 413 | 413 | 413 | ITT OS and as-treated safety populations had the same N. |
| KEYNOTE-394 | 453 | 453 | 453 | 452 | 453 | 452 | Safety N excludes 1 untreated patient. |
| REACH | 565 | 565 | 565 | 553 | — | — | Full-ITT OS/KM and full safety; no MDC because OS was nonsignificant. |
| REACH-2 | 292 | — | — | — | — | — | Included in the AFP ≥400 ng/mL subgroup analysis only; not included in the primary post-TKI network. |
| RELIVE | 397 | 397 | 397 | — | — | — | No eligible grade ≥3 AE count. |
| RESORCE | 573 | 573 | 573 | 567 | 573 | 567 | Safety N excludes 6 untreated patients. |
| S-CUBE | 334 | 333 | 333 | — | — | — | KM N = 333; no eligible grade ≥3 AE count. |
| TOTAL | 5505 | 5000 | 5000 | 3413 | 1934 | 1925 | Analysis-specific totals. |

Note: N is analysis-specific. An em dash (—) indicates no primary OS, KM, safety, or MDC contribution. REACH used full-ITT OS/KM and full-safety data. REACH-2 full ITT contributed only to the AFP ≥400 ng/mL analysis. MDC used unrounded full-network P-scores.

**Table S6. Common-effects versus random-effects sensitivity analysis of overall survival after prior TKI therapy.**

| Treatment | Reference | Random-effects HR (95% CI) | Common-effects HR (95% CI) | Direction consistent |
| --- | --- | --- | --- | --- |
| ADI-PEG20 | Placebo/BSC | 1.02 (0.85–1.23) | 1.02 (0.85–1.23) | Yes |
| Brivanib | Placebo/BSC | 0.89 (0.69–1.15) | 0.89 (0.69–1.15) | Yes |
| Cabozantinib | Placebo/BSC | 0.70 (0.55–0.89) | 0.70 (0.55–0.89) | Yes |
| Everolimus | Placebo/BSC | 1.05 (0.86–1.28) | 1.05 (0.86–1.28) | Yes |
| Tivantinib | Placebo/BSC | 0.82 (0.58–1.15) | 0.82 (0.58–1.15) | Yes |
| Pembrolizumab | Placebo/BSC | 0.79 (0.67–0.93) | 0.79 (0.67–0.93) | Yes |
| Ramucirumab | Placebo/BSC | 0.87 (0.72–1.05) | 0.87 (0.72–1.05) | Yes |
| Dox-NPs | Placebo/BSC | 1.00 (0.78–1.28) | 1.00 (0.78–1.28) | Yes |
| Regorafenib | Placebo/BSC | 0.63 (0.50–0.79) | 0.63 (0.50–0.79) | Yes |
| S-1 | Placebo/BSC | 0.86 (0.67–1.10) | 0.86 (0.67–1.10) | Yes |

Note: Trial-reported HRs were synthesized for the post-TKI overall-survival network. The random-effects model was used for the primary analysis, and the common-effects model was fitted as a sensitivity analysis. HRs <1 favor the listed treatment over placebo/BSC. Effect directions and statistical significance were unchanged across models; identical estimates reflect estimated between-study heterogeneity of τ²=0. BSC, best supportive care; CI, confidence interval; HR, hazard ratio; OS, overall survival; TKI, tyrosine kinase inhibitor.

**Table S7. Sensitivity analyses of safety heterogeneity and the exploratory MDC analysis.**

| **Panel A. Study-level heterogeneity diagnostics for the safety network** | | | | | | |
| --- | --- | --- | --- | --- | --- | --- |
| Study | Treatment | Direct OR (95% CI) | Q contribution, % | Residual df after exclusion | τ² after exclusion | I² after exclusion, % |
| KEYNOTE-240 | Pembrolizumab | 1.29 (0.86–1.95) | 50.73 | 0 | NE | NE |
| KEYNOTE-394 | Pembrolizumab | 2.28 (1.52–3.42) | 49.27 | 0 | NE | NE |
| BRISK-PS | Brivanib | 3.24 (2.09–5.02) | 0.00 | 1 | 0.1164 | 72.68 |
| CELESTIAL sorafenib-only subgroup | Cabozantinib | 4.73 (3.16–7.08) | 0.00 | 1 | 0.1164 | 72.68 |
| EVOLVE-1 | Everolimus | 2.23 (1.54–3.23) | 0.00 | 1 | 0.1164 | 72.68 |
| RESORCE | Regorafenib | 3.10 (2.16–4.44) | 0.00 | 1 | 0.1164 | 72.68 |
| REACH | Ramucirumab | 1.79 (1.27–2.51) | 0.00 | 1 | 0.1164 | 72.68 |

| **Panel B. Leave-one-out sensitivity analyses of the safety network** | | | | | | |
| --- | --- | --- | --- | --- | --- | --- |
| Scenario | Treatment | OR (95% CI) | Safety P-score | Safety rank | τ² | I², % |
| Primary | Pembrolizumab | 1.72 (0.99–2.99) | 0.673 | 2 | 0.1164 | 72.68 |
| Primary | Regorafenib | 3.10 (1.45–6.62) | 0.309 | 5 | 0.1164 | 72.68 |
| Primary | Cabozantinib | 4.73 (2.16–10.32) | 0.104 | 7 | 0.1164 | 72.68 |
| Excluding KEYNOTE-240 | Pembrolizumab | 2.28 (1.52–3.42) | 0.565 | 4 | NE | NE |
| Excluding KEYNOTE-240 | Regorafenib | 3.10 (2.16–4.44) | 0.293 | 5 | NE | NE |
| Excluding KEYNOTE-240 | Cabozantinib | 4.73 (3.16–7.08) | 0.030 | 7 | NE | NE |
| Excluding KEYNOTE-394 | Pembrolizumab | 1.29 (0.86–1.95) | 0.827 | 2 | NE | NE |
| Excluding KEYNOTE-394 | Regorafenib | 3.10 (2.16–4.44) | 0.270 | 5 | NE | NE |
| Excluding KEYNOTE-394 | Cabozantinib | 4.73 (3.16–7.08) | 0.029 | 7 | NE | NE |

| **Panel C. Sensitivity analyses of the exploratory MDC assessment** | | | | | |
| --- | --- | --- | --- | --- | --- |
| Scenario | OS:safety weight | Pembrolizumab MDC | Regorafenib MDC | Cabozantinib MDC | MDC ordering |
| Primary | 1:1 | 0.439 | 0.693 | 0.908 | Pembrolizumab (1st) → Regorafenib (2nd) → Cabozantinib (3rd) |
| Excluding KEYNOTE-240 | 1:1 | 0.524 | 0.709 | 0.981 | Pembrolizumab (1st) → Regorafenib (2nd) → Cabozantinib (3rd) |
| Excluding KEYNOTE-394 | 1:1 | 0.340 | 0.731 | 0.982 | Pembrolizumab (1st) → Regorafenib (2nd) → Cabozantinib (3rd) |
| Efficacy-prioritized | 2:1 | 0.431 | 0.567 | 0.752 | Pembrolizumab (1st) → Regorafenib (2nd) → Cabozantinib (3rd) |
| Safety-prioritized | 1:2 | 0.447 | 0.799 | 1.042 | Pembrolizumab (1st) → Regorafenib (2nd) → Cabozantinib (3rd) |

Note: The primary safety analysis used a random-effects NMA. KEYNOTE-240 and KEYNOTE-394 were the only replicated direct comparison and accounted for the estimable residual heterogeneity. After exclusion of either study, residual df=0 and heterogeneity was no longer estimable; common-effect estimates were therefore used in these sensitivity analyses. Alternative MDC weights of 2:1 and 1:2 were exploratory and do not represent validated clinical preference weights. No validated clinically meaningful threshold for MDC differences is available.

**Figure S7. Trial-reported versus reconstructed overall-survival HRs.**

Note: Blue squares show trial-reported HRs and gray diamonds show reconstructed HRs; horizontal bars indicate 95% CIs. Trial-reported HRs were used in the primary random-effects NMA. The REACH full ITT population is included with a matched reconstruction, whereas REACH-2 is excluded. Reconstructed estimates are descriptive comparisons only and did not enter the formal NMA, P-score calculation, or treatment ranking. CI, confidence interval; HR, hazard ratio; ITT, intention-to-treat; NMA, network meta-analysis.

**Figure S8. Node-splitting assessment in the post-immune-checkpoint-inhibitor network.**

The observational post-ICI network contained one closed loop connecting lenvatinib, sorafenib, and regorafenib. Ratios of HRs compare direct with indirect estimates. No statistically detectable disagreement was observed (all p=0.702), but the assessment included few studies and had limited power; it does not establish consistency. HR, hazard ratio; ICI, immune checkpoint inhibitor.
